# Comparison of Self-harm or Overdose Among Adolescents and Young Adults Before vs During the COVID-19 Pandemic in Ontario

**DOI:** 10.1001/jamanetworkopen.2021.43144

**Published:** 2022-01-12

**Authors:** Joel G. Ray, Peter C. Austin, Kayvan Aflaki, Astrid Guttmann, Alison L. Park

**Affiliations:** 1 Departments of Medicine and Obstetrics and Gynaecology, St Michael’s Hospital,; 2 Institute of Health Policy, Management and Evaluation, University of Toronto, Toronto, Ontario, Canada; 3 ICES, Toronto, Ontario, Canada; 4 Institute of Medical Science, University of Toronto, Toronto, Ontario, Canada; 5 Hospital for Sick Children, Department of Paediatrics, Institute of Health Policy, Management and Evaluation, Dalla Lana School of Public Health, Edwin S.H. Leong Centre for Healthy Children, University of Toronto, Toronto, Ontario, Canada

## Abstract

**Importance:**

Self-harm and deaths among adolescents and young adults are notably related to drug poisonings and suicide. With the emergence of the COVID-19 pandemic, there are projections about a greater likelihood of such events arising among adolescents and young adults.

**Objective:**

To evaluate the risk of self-harm, overdose, and all-cause mortality among adolescents and young adults during the COVID-19 pandemic.

**Design, Setting, and Participants:**

This population-based cohort study took place in Ontario, Canada, where a universal health care system captures all emergency department (ED) visits, hospitalizations, and deaths. The participants included all adolescents and young adults born in Ontario between 1990 and 2006, who were aged 14 to 24 years between March 1, 2018, and June 30, 2021.

**Exposures:**

The COVID-19 pandemic era (April 1, 2020 to June 30, 2021), relative to the 2 years preceding the pandemic (March 1, 2018 to February 28, 2020).

**Main Outcomes and Measures:**

ED encounters or hospitalizations for self-harm or overdose. A secondary outcome was self-harm, overdose, or all-cause mortality. Cause-specific hazard models to estimate hazard ratios (HR) and 95% CIs were used for the primary outcome. Follow-up started at March 1, 2018, or the individual’s 14th birthday, whichever was later, and age was used as the time scale.

**Results:**

In this study, 1 690 733 adolescents and young adults (823 904 [51.3%] female participants) were included with a median (IQR) age of 17.7 (14.1-21.4) years at start of follow-up. After 4 110 903 person-years of follow-up, 6224 adolescents and young adults experienced the primary outcome of self-harm or overdose during the pandemic (39.7 per 10 000 person-years) vs 12 970 (51.0 per 10 000 person-years) prepandemic, with an HR of 0.78 (95% CI, 0.75-0.80). The risk of self-harm, overdose, or death was also lower during than before the pandemic (HR, 0.78; 95% CI, 0.76-0.81), but not all-cause mortality (HR, 0.95; 95% CI, 0.86-1.05).

**Conclusions and Relevance:**

Among adolescents and young adults, the initial 15-month period of the COVID-19 pandemic was associated with a relative decline in hospital care for self-harm or overdose.

## Key Points

**Question:**

What was the risk of self-harm or overdose among adolescents and young adults in Ontario, Canada, during the SARS-CoV-2 pandemic compared with the 2 years preceding?

**Findings:**

In this cohort study of 1 690 733 adolescents and young adults in all of Ontario, the rate of self-harm or overdose was lower during (39.7 per 10 000 person-years) than before (51.0 per 10 000 person-years) the pandemic.

**Meaning:**

At least up to mid-2021, the COVID-19 pandemic has not been associated with an excess of self-harm requiring health care among adolescents and young adults.

## Teaser

This cohort study evaluates the risk of self-harm and overdose among adolescents and young adults during vs before the COVID-19 pandemic.

## Introduction

Within North America, self-harm and deaths among adolescents and young adults aged 14 to 24 years remain prevalent and are notably related to drug poisonings and suicide.^1,2,3,4,5^ The risk of premature death increases around age 14 years, especially among males, rural residents,^4^ and those who exhibit persistently high despair scores—so-called deaths of despair.^6^

With the emergence of the COVID-19 pandemic, a high rate of suicidal thoughts, severe depression, and anxiety was seen among university and elementary students during quarantine.^7,8^ Although there are projections about the greater likelihood of suicide^9^ and deaths of despair from the COVID-19 pandemic,^10^ actual data are lacking, especially among adolescents and young adults. In late 2000, John et al correctly asserted that “[s]upposition, however, is no replacement for evidence. Timely data on rates of suicide are vital.”^11^ This is especially important among young people. The current study, which was completed within a universal health care system that captures all emergency department (ED) visits, hospitalizations, and deaths, evaluated the risk of self-harm, overdose, and all-cause mortality among adolescents and young adults during vs before the COVID-19 pandemic.^11^

## Methods

This population-based dynamic cohort study used data authorized under section 45 of Ontario’s Personal Health Information Protection Act (PHIPA) and does not require review by a research ethics board. This study followed the Strengthening the Reporting of Observational Studies in Epidemiology (STROBE) reporting guideline for cohort studies.

This study began with all hospital births in the province of Ontario, Canada, 1990 to 2006, and in which the child survived to age 1 year, as described elsewhere.^4^ The current study specifically included those who would be 14 years or older by the end of the study period (June 30, 2021), but younger than 25 years on March 1, 2018 (the beginning date of the prepandemic period). All data sets were linked using unique encoded identifiers and analyzed at ICES (eTable in the Supplement). ICES is an independent, not-for-profit organization that houses diagnostic, procedural, and sociodemographic data for Ontario residents. Data are linked deterministically, including hospitalizations, ED visits, census data, births, and deaths (eTable in the Supplement).

As a true dynamic cohort, persons could enter and exit the observation period according to their age eligibility. Moreover, a cohort member could exit the study by censoring or having an outcome event, as described below. The primary composite outcome was an ED encounter or hospitalization for self-harm or overdose or poisoning of accidental or unknown intent—the latter was included consideringthat more than 50% of poisonings among youth presenting to hospital are intentional (eTable in the Supplement).^12^ A secondary composite outcome was self-harm, overdose, or all-cause mortality (the latter to capture out-of-hospital deaths). All deaths in the province, within the study period, were ascertained using the Registered Persons Database, which contains demographic information and encrypted health care numbers for all residents of Ontario (eTable in the Supplement).

### Statistical Analysis

Time-to-event survival analysis was performed using cause-specific hazard models to estimate hazard ratios (HRs) and 95% CIs of the primary outcome after accounting for the competing risk of death. A conventional Cox model was used for the secondary outcome. For each participant, the start of follow-up (time 0) was at the adolescent or young adult’s 14th birthday or March 1, 2018, whichever was later, and age was used as the time scale. Left truncation starting at age 14 years ensured that participants were assessed for the outcome only when they were actually under follow-up by the study.^13^ Death was treated as a competing risk, except for the secondary composite and the all-cause mortality outcomes, and participants were censored at the end of provincial health coverage, which might occur with migration out of the province, age 25 years, or end of the study period on June 30, 2021.

March 2020 was a transitional period during which the pandemic gradually emerged in Ontario, and school closures were announced across the province. Therefore, the exposure variable was treated as a 3-level time-varying covariate: (1) prepandemic period (March 1, 2018, to February 28, 2020); (2) the transitional period of March 1 to 31, 2020 (omitted herein); and (3) pandemic period (April 1, 2020, to June 30, 2021). The proportional hazards assumption was tested as an interaction between time (ie, age) and the COVID-19 pandemic exposure.

For the primary composite outcome, we additionally analyzed whether the HR differed by important factors associated with premature mortality,^4^ namely (1) male vs female sex, (2) income quintiles 1 to 2 (lower) vs 3 to 5 (higher), (3) rural vs urban residence, and (4) by age groups 14 to 17 years vs 18 to 24 years during the observation period.

We also restricted the primary composite outcome to self-harm or overdose with a hospital admission, assuming that they might be more severe in nature. Finally, we analyzed the individual component outcomes of self-harm, overdose, and all-cause mortality. Statistical significance was set at a 2-sided *P* value of less than .05. Statistical analyses were done using SAS, version 9.4 for UNIX (SAS Institute), from October 2021 to November 2021.

## Results

In this study, 1 690 733 adolescents and young adults were included in the final cohort (eFigure in the Supplement). Their median age at the start of follow-up was 17.7 (IQR, 14.1-21.4) years and 21.0 (IQR, 17.3-24.7) years at the end of follow-up (Table). In this study, 823 904 (48.7) were female, 587 547 (34.8%) resided in the lowest 2 income quintile neighborhoods, 176 024 (10.4%) were rural residents, and 26 410 (1.6%) had a history of self-harm or overdose at time 0.

**Table.** Description of 1 690 733 Adolescents and Young Adults Born in Ontario, Canada, Between 1990 and 2016, and Who Were Aged 14 to 24 Years Between March 1, 2018, and June 30, 2021

Characteristic	Participant, No. (%) (N = 1 690 773)
Sex	
Female	823 904 (48.7)
Male	866 829 (51.3)
Residential income quintile at time 0	
1 (lowest)	285 357 (16.9)
2	302 190 (17.9)
3	333 626 (19.7)
4	361 674 (21.4)
5 (highest)	401 454 (23.7)
Unknown	6432 (0.4)
Rural residence at time 0	
Rural	176 024 (10.4)
Unknown	5677 (0.3)
History of self-harm or overdose before time 0	26 410 (1.6)
Age, median (IQR), y	
At time 0	17.7 (14.1-21.4)
At the start of the COVID-19 pandemic	19.8 (16.2-23.5)
At end of study follow-up	21.0 (17.3-24.7)

**Figure 1.**
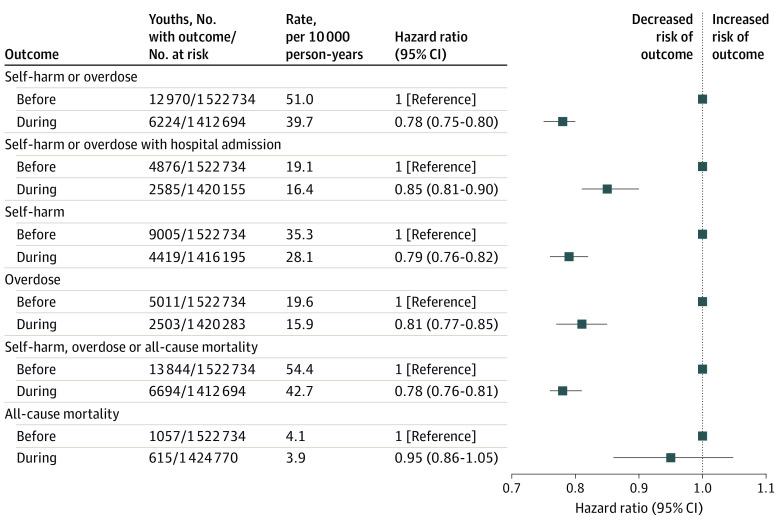
Risk of Adolescent and Young Adult (Age 14 to 24 Years) Self-harm, Overdose, and All-Cause Mortality Before and During the COVID-19 Pandemic The risk of self-harm, overdose, and all-cause mortality was assessed individually and with hospital admission and compared adolescents and young adults aged 14 to 24 years during the COVID-19 pandemic (April 1, 2020, to June 30, 2021) vs the 2 preceding years (March 1, 2018, to February 28, 2020). Self-harm, overdose, and all-cause mortality were censored on death, loss of Ontario Health Insurance Plan eligibility, turning 25 years old, or arriving at the end of the study period of June 30, 2021. During the omitted period of March 1 to 31, 2020, 397 self-harm or overdose events occurred, or 37.3 per 10 000 person-years.

After 4 110 903 person-years of follow-up, 6224 adolescents and young adults experienced the primary outcome of self-harm or overdose during the pandemic (39.7 per 10 000 person-years) vs 12 970 (51.0 per 10 000 person-years) prepandemic (HR, 0.78; 95% CI, 0.75-0.80) (Figure 1). The risk of self-harm or overdose requiring hospital admission was also lower during the pandemic (HR, 0.85; 95% CI, 0.81-0.90). Of note, during the omitted transitional period of March 1 to 31, 2020, there were 397 self-harm or overdose events, which is equivalent to a rate of 37.3 per 10 000 person-years.

The risk of the secondary outcome of self-harm, overdose, or death was also lower than before the pandemic (HR 0.78; 95% CI, 0.76-0.81) (Figure 1). During the pandemic, self-harm was the most common component outcome (28.1 per 10 000 person-years), followed by overdose (15.9 per 10 000 person-years), and then death (3.9 per 10 000 person-years). Of the individual component outcomes, only the risk of death did not change from before to during the pandemic (HR 0.95; 95% CI, 0.86-1.05) (Figure 1).

**Figure 2.**
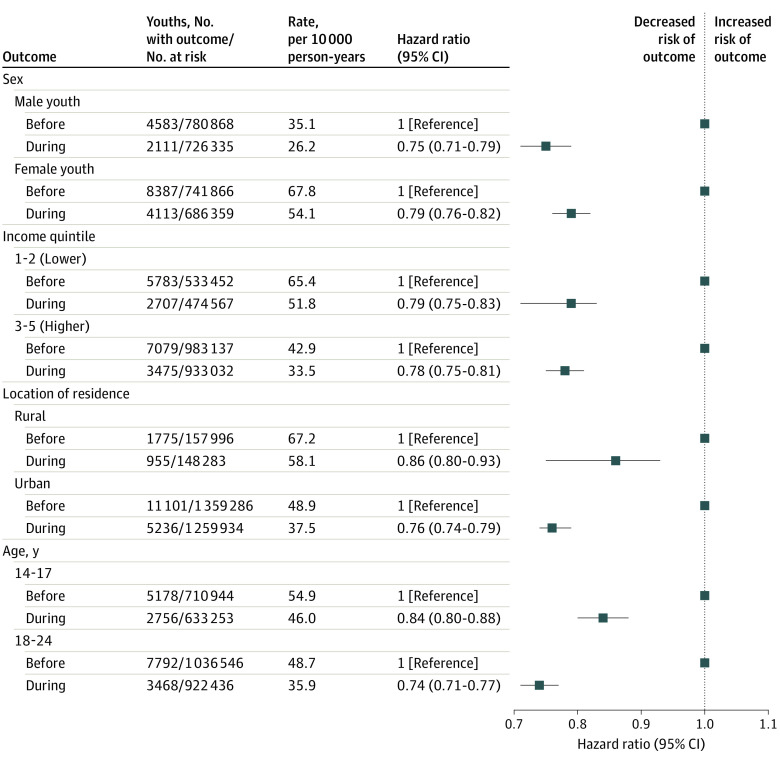
Risk of Adolescent and Young Adult (Age 14 to 24 Years) Self-harm and Overdose Before and During the COVID-19 Pandemic Stratified by Sex, Residential Income Quintile, Rural or Urban Residence, and Age Group Self-harm and overdose were censored upon death, loss of Ontario Health Insurance Plan eligibility, attained age 25 years, or arrival at the end of the study period of June 30, 2021.

The lower HR for the primary outcome persisted upon stratification by sex, income quintile, or rurality, although the absolute events rates were higher for females, low-income residents, and rural residents (Figure 2). In addition, the lower HR for the primary outcome was more pronounced among adolescents and young adults aged 18 to 24 years (0.74; 95% CI, 0.71-0.77) than those aged 14 to 17 years (0.84; 95% CI, 0.80-0.88) (Figure 2).

## Discussion

Among adolescents and young adults, a 15-month period of the COVID-19 pandemic was associated with a relative decline in self-harm or overdose. The robust nature of the current findings suggests that, at least up to the middle of 2021, the COVID-19 pandemic has not led to an excess of intentional injury among adolescents and young adults. This is contrary to the perceived expectations of others,^11^ and supports the findings during the first 6 months of the pandemic in Austria, in which there was a relative decline in suicide among all adults.^14^ While internet searches suggestive of acute anxiety rose during the early part of the pandemic, they subsequently returned to prepandemic levels.^15^ Among 2 large tertiary pediatric hospitals in Quebec, Canada, a study conducted between January 2018 and December 2020 observed no prepandemic vs pandemic changes in ED visits for substance use or suicide.^16^ In a single-center study^17^ from Philadelphia during that same era, comprising 5 to 24-year-olds, the mean number of monthly ED mental health visits significantly fell during the COVID-19 pandemic, even though the proportion of all ED visits for a mental health condition increased from 4.0% to 5.7%. Similar findings were seen in a cross-sectional study of all ED visits within 27 US pediatric hospitals during the pandemic (March to August 2020) vs the prepandemic period.^18^ Our current findings expand on these studies, providing not only an extended period of assessment of the pandemic up to June 2021, but a specific focus on 14 to 24-year-olds, who are susceptible to self-harm and overdose, which were events more likely to be captured by an ED encounter or a death.^4,6^ Even so, it must be reiterated that some fatal or nonfatal cases of self-harm or overdose may have been missed because of a lack of presentation to an ED, or incomplete capture of either as a fatality.

In their late 2020 publication, John et al^11^ called for more high-quality evidence regarding the association of the COVID-19 pandemic with suicide rates. Since then, their systematic review of 78 eligible studies published up to October 2020—14% of which specifically focused on adolescents and young adults, and many not yet peer-reviewed—found no evidence of a rise in suicide after onset of the pandemic.^19^ Even so, they documented relative declines in presentation to ED for suicidal behavior and possibly greater suicidal thoughts. Indeed, ongoing surveillance in various jurisdictions might see the emergence of a different pattern, and other mental health sequelae, among adolescents and young adults.

### Limitations

This study has limitations. Because our data did not capture self-harm or overdose events without a hospital or ED encounter, less life-threatening cases may have been missed. One analysis was confined to self-harm and overdose cases requiring hospital admission, which was less frequent during than before the pandemic. We lacked details about completed suicides occurring out-of-hospital, but we captured all-cause death, which was relatively uncommon. For example, during the pandemic, there were 3.9 deaths per 10 000 person-years compared with 28.1 self-harm events per 10 000 person-years. Mortality did not change from before to during the pandemic, because most self-harm events among adolescents and young adults tend to be nonfatal,^20^ while the case-fatality rate from COVID-19 in this age group has been very low.^21^ The overall large number of cases recorded within a universal health care system enabled us to generate stable and precise risk estimates, including by sociodemographic factors related to intentional injury or mortality.^4^

## Conclusions

In this study, there was a relative decline in hospital encounters for self-harm or overdose among adolescents and young adults during the first 15 months of the COVID-19 pandemic. It should be determined if this phenomenon continued within subsequent waves of the pandemic, or if unrealized self-harm or overdose events have occurred outside of a hospital setting.

## Notes

Supplement. **eTable.** Cohort Entry and Exclusion Criteria, Methods, and Coding to Identify Study Outcomes **eFigure.** Flow Diagram for Creation of the Study Cohort
