# Supplementary material for: Comparison of Self-harm or Overdose Among Adolescents and Young Adults Before vs During the COVID-19 Pandemic in Ontario
Source: JAMA Netw Open. 2022 Jan 12;5(1):e2143144. doi: 10.1001/jamanetworkopen.2021.43144 (PMC8756304; doi:10.1001/jamanetworkopen.2021.43144)
Supplement: Supplement. — eTable. Cohort Entry and Exclusion Criteria, Methods, and Coding to Identify Study Outcomes eFigure. Flow Diagram for Creation of the Study Cohort [file jamanetwopen-e2143144-s001.pdf]

Supplemental Online Content

Ray JG, Austin PC, Aflaki K, Guttmann A, Park AL. Comparison of self-harm or overdose among adolescents and young adults before vs during the COVID-19 pandemic in Ontario. *JAMA Netw Open*. 2022;5(1):e2143144. doi:10.1001/jamanetworkopen.2021.43144

eTable. Cohort Entry and Exclusion Criteria, Methods, and Coding to Identify Study Outcomes
eFigure. Flow Diagram for Creation of the Study Cohort

This supplemental material has been provided by the authors to give readers additional information about their work.

© 2022 Ray JG et al. *JAMA Network Open.*

eTable 1. Cohort Entry and Exclusion Criteria, Methods and Coding to Identify Study Outcomes

Assessment	Timing	Disease, procedure or condition	ICD-10-CA diagnostic codes in CIHI-DAD and NACRS	Other sources
Inclusion criteria	At the time of birth	Obstetrical live birth delivery in Ontario, Canada, between April 1, 1993 and March 31, 2006	MOMBABY (links the hospital admission records of delivering mothers and newborns in CIHI-DAD)	--
Exclusion criteria	At the time of birth	a. Invalid maternal or youth healthcare number<br>b. Invalid/missing maternal or youth age, sex, or birth date<br>c. Invalid maternal death date (before delivery date)<br>d. Maternal age < 16 or > 50 years<br>e. Gestational weeks at birth is < 20 weeks or missing	MOMBABY	RPDB (contains demographic information & encrypted healthcare numbers for all individuals eligible for OHIP)
	March 1, 2018	Youth's age ≥ 25 years at the start of follow-up	--	RPDB
	June 30, 2021	Youth's age < 14 years at the end of follow-up	--	RPDB
	March 1, 2018 or 14th birthday – whichever is later	Youth at the start of follow-up:<br>a. Not alive<br>b. Not eligible for OHIP<br>c. Not an Ontario resident	--	RPDB
Main exposure	March 1, 2018 to June 30, 2021	3-level time-varying SARS-CoV-2 pandemic: (1) pre-pandemic period (March 1, 2018 to February 28, 2020); (2) transition period (March 1 to 31, 2020); (3) pandemic period (April 1, 2020 to June 30, 2021)	--	--
Main outcome	March 1, 2018 to June 30, 2021 (except for March 1 to March 31, 2020)	Intentional injury defined as emergency department visit or hospitalization for self-harm or poisoning/overdose of accidental or unknown intent, of the youth at age 14 to 24 years	Self-harm: X60-X84<br>Poisoning/overdose: X40-X42, X46, X47, Y10-Y19	--
Secondary outcomes	Same	All-cause mortality or intentional injury defined as emergency department visit or hospitalization for self-harm, poisoning/overdose of accidental or unknown intent, of the youth at age 14 to 24 years	Self-harm: X60-X84<br>Poisoning/overdose: X40-X42, X46, X47, Y10-Y19	All-cause mortality: RPDB

© 2022 Ray JG et al. *JAMA Network Open.*

eTable 1 Continued. Cohort Entry and Exclusion Criteria, Methods and Coding to Identify Study Outcomes

Assessment	Timing	Disease, procedure or condition	ICD-10-CA diagnostic codes in CIHI-DAD and NACRS	Other sources
	Same	Type of injury or death of the youth at age 14 to 24 years:<br>a. Intentional self-harm<br>b. Poisoning or overdose<br>c. All-cause mortality	Self-harm: X60-X84<br>Poisoning/overdose: X40-X42, X46, X47, Y10-Y19	All-cause mortality: RPDB
	Same	Intentional self-harm or poisoning/overdose with a hospital admission of the youth at age 14 to 24 years	Hospital admission (direct or from an emergency department) with one the following:<br>Self-harm: X60-X84<br>Poisoning/overdose: X40-X42, X46, X47, Y10-Y19	--
Censoring variables	Starting at March 1, 2018 or the youth's 14th birthday – whichever is later	Death of the youth	--	RPDB
	Same	Youth's 25th birthday	--	RPDB
	Same	Loss of OHIP eligibility of the youth	--	RPDB
	June 30, 2021	End of the study period	--	--
Stratification variables	At the time of birth	Youth sex (male, female)	--	RPDB
	March 1, 2018 or 14th birthday – whichever is later	Low area-level income quintile at the start of follow-up (quintile 1-2 vs. quintile 3-5)	--	Statistics Canada census data
	Same	Rural residency of the youth (rural vs. urban)	--	Statistics Canada census data
	March 1, 2018 to June 30, 2021	Age 14-17 years vs. 18-24 years	--	RPDB

Abbreviations: CIHI: Canadian Institute for Health Information; DAD: Discharge Abstract Database; ICD-10-CA: International Classification of Diseases, 10th Revision, Canada; OHIP: Ontario Health Insurance Plan; NACRS: National Ambulatory Care Reporting System; SDS: Same Day Surgery Database; RPDB: Registered Persons Database

© 2022 Ray JG et al. *JAMA Network Open.*

eFigure 1. Flow Diagram for Creation of the Study Cohort

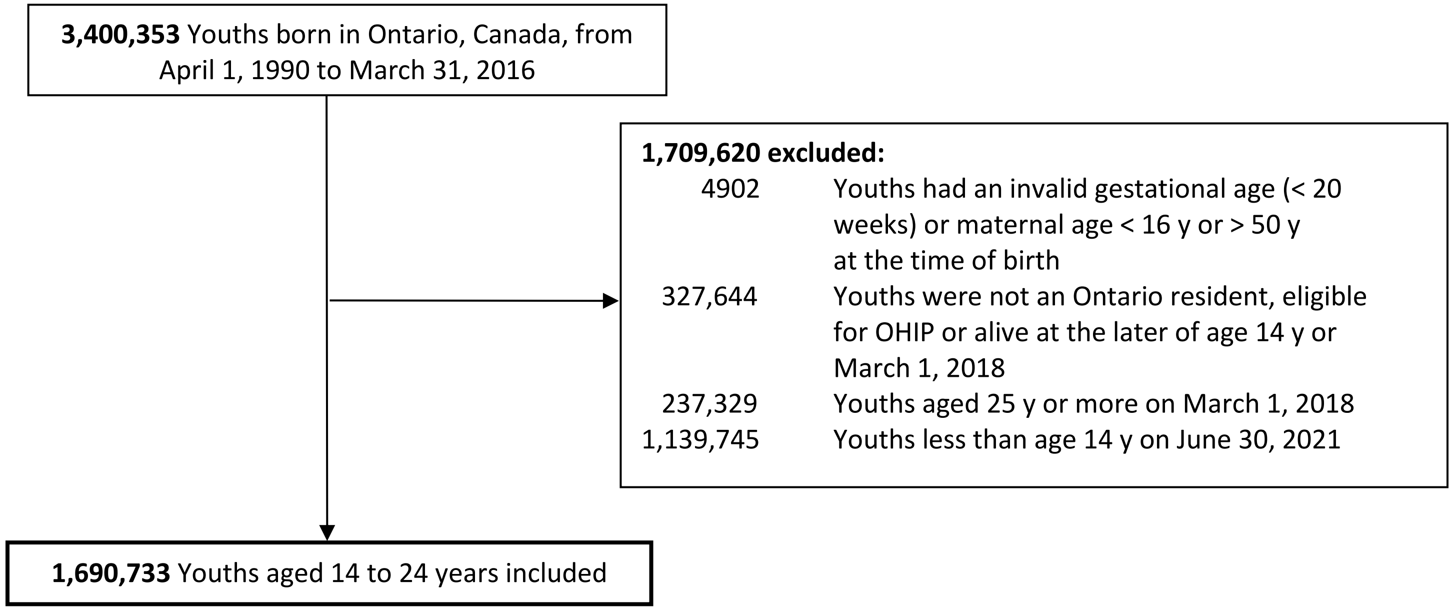

© 2022 Ray JG et al. *JAMA Network Open.*
